# A context-responsive health systems intervention improves the uptake of early infant HIV diagnosis: Controlled before and after study in Malawi

**DOI:** 10.1371/journal.pgph.0006269

**Published:** 2026-04-21

**Authors:** Leticia Chimwemwe Suwedi-Kapesa, Augustine Choko, James Chirombo, Dorcus Nothale, Nicola Desmond, Angela Obasi

**Affiliations:** 1 Department of International Public Health, Liverpool School of Tropical Medicine, Liverpool, England, United Kingdom; 2 Public Health Research group, Malawi-Liverpool-Wellcome Trust Clinical Research Programme, Blantyre, Malawi; 3 Department of Public Health Surveillance and Disease Intelligence, Public Health Institute of Malawi, Lilongwe, Malawi; 4 Department of Nursing, Blantyre District Health Office, Malawi; 5 Department of Global Health and Development, London School of Hygiene and Tropical Medicine, London, United Kingdom; 6 Axess Sexual Health, Liverpool University Hospitals NHS Foundation Trust, Liverpool, United Kingdom; NYU Grossman School of Medicine: New York University School of Medicine, UNITED STATES OF AMERICA

## Abstract

Health system challenges limit uptake of early infant diagnosis of HIV (EID). Context-appropriate strategies are required to achieve global 95% six-week testing target. We evaluated a co-designed context-appropriate enhanced health system (EEHS) intervention to strengthen client identification, appointment booking systems, leadership, and facility-based training to improve enrolment of Infants Exposed to HIV (IEH) in HIV care and testing at six weeks. We conducted a before-after intervention evaluation (15 October 2022–5 June 2023) at one urban and rural primary health facilities in Blantyre, Malawi. During pre-intervention period (15 October 2022–18 January 2023), women received standard-of-care EID services. In post-intervention period (19 January to 5 June 2023), women received EID services with EEHS intervention. Data was extracted for women living with HIV and IEH at birth to six weeks. Outcomes were proportion of IEH tested at six weeks (primary) and enrolled in HIV care at birth (Secondary). Logistic regression models were fitted to compute odds ratios (ORs) and 95% confidence intervals (CI). We enrolled 60 women with IEH: 11/60 (18.3%) in rural and 11/60 (18.3%) urban before intervention versus 6/60 (10%) in rural and 54/60 (90%) in urban post-intervention. Median age was 27.5 (interquartile range (IQR), 23–31) pre-intervention and 28 (IQR, 23–32) post-intervention. Six-weeks HIV testing of IEH improved post intervention versus pre-intervention from 46/58 (79%) to 43/46 (93%) (OR 3.74, 95% CI: 1.10-17.23; p = 0.052), with a statistically significant association in adjusted analysis (aOR 4.35, 95% CI: 1.21–21.25) p = 0.038). Enrolment of IEH in HIV care at birth post-intervention increased from 47/60 (78%) to 55/60 (92%) (OR 3.04: 95% CI 1.06-10.06, p = 0.048), with a statistically significant association in adjusted analysis (aOR 3.33: 95% CI 1.13-11.25, p = 0.036). EEHS intervention, potentially improves IEHs’ enrolment in HIV care and six-weeks HIV testing, addressing health system challenges, however it requires validation through randomised studies.

## Introduction

HIV testing at two months for infants exposed to HIV has increased from 50% in 2015 to 67% in 2023 [1], yet the 95% global targets remain unmet, making achieving the goal of an HIV-free generation by 2030 unlikely [2]. Fifty per cent of children with HIV die before two years if not on treatment [1], making early infant diagnosis of HIV (EID) a priority to prevent high morbidity and mortality among infants living with HIV. The 2021 World Health Organisation (WHO) recommends early enrolment of infants exposed to HIV in HIV care programs for monitoring and provision of prophylaxis according to HIV risk exposure status. The WHO recommends that HIV tests should be offered to children below 18 months through point-of-care (POC) from 4-6 weeks using Deoxyribonucleic acid (DNA) polymerase chain reaction (PCR) [3].

From 2021 to 2023, infant HIV testing rates in eastern and southern Africa increased from 77% to 80% respectively [2]. Among other interventions, such as peer mentorship, the implementation of POC testing of HIV has been instrumental in increasing these rates [2]. In Malawi, the testing rate has risen from 22% in 2015 to 85% in 2023 [4]. However, delays and missed opportunities caused by health system factors such as limited resources, incomplete service integration, stigma, poor communication, long waiting times for women and infants to receive all required services, and the limited capacity and lengthy processing times of the POC machine hinder the full implementation of WHO recommendations [2,5,6].

Understanding variable contexts in health facilities is key to supporting the effective implementation of guidelines and interventions to address health system limitations [7]. Suwedi-Kapesa et al [5] analysed context-specific challenges to EID services in two primary health facilities in Blantyre, Malawi, a district with a high (10.9%) prevalence of HIV among pregnant women [8]. The findings showed that 32% (39/163) of infants exposed to HIV born at study sites in 2018 were enrolled in HIV care at birth, and 52% (85/163) were tested at six weeks [5]. Due to specific contextual factors within urban and rural facilities, implementation gaps and delays limited the delivery of patient-centred integrated services [5]. These included a lack of POC machines at rural facilities, the inability of POC machines to process more tests at one time, each taking one hour to process, starting late to offer POC HIV testing, limited infrastructure leading to limited service days, overburdened spaces and failure to schedule clinic appointments for mother and infant pairs together [5,9].

In response, Suwedi Kapesa et al co-designed a context-responsive EEHS intervention to improve client identification, delivery of client-centred service integration, enhanced health care worker (HCW) coordination, accountability and strengthened capacity building for optimal service delivery to improve enrolment of infants in HIV care clinics at birth and HIV testing at six weeks to meet the WHO recommendations [9]. Suwedi Kapesa et al. used the Power Matrix Index to identify key stakeholders. They employed the Behaviour Change Wheel and the Theoretical Domains Framework to guide the development of the intervention and the consolidated framework construct in healthcare, which informed planning for sustainability, as discussed in their publication [9]. This paper presents findings from a controlled before and after evaluation of the impact of the EEHS intervention on uptake of EID services from birth to six weeks in two primary health facilities in Blantyre, District, Southern Malawi.

## Materials and methods

### Ethics statement

The Liverpool School of Tropical Medicine (22–025) and the College of Medicine (Local) Research & Ethics Committee (P.11/23–0438) approved this study. Data extraction did not require informed consent, as no identifying information was extracted. The director of health and social services for Blantyre district and the research team provided written approval to conduct the study.

### Inclusivity in global research

Additional information regarding the ethical, cultural and scientific considerations specific to inclusivity in global research is included in supporting information (S1 Checklist)

### Study setting

Blantyre is one of the districts with a high HIV prevalence among pregnant women at 10.9% [8]. Building on the study by Suwedi Kapesa et al., which assessed EID service delivery at two primary government health facilities in Blantyre, Malawi, we selected the same two sites to evaluate the intervention [5]. Briefly, Blantyre had 28 government-owned facilities (9 urban, 19 rural). Four used Abbott mPima POC machines; four had Cepheid GeneXpert machines for TB testing, viral load assessment, and EID [10]. One of the four GeneXpert facilities served as a hub for centralised testing for the other facilities, referred to as near-POC testing [10]. The Suwedi Kapesa et al. 2024 study aimed to conduct research at two sites to gain an in-depth understanding of the context [5]. The study sites were purposively selected to include 1) facilities with and without POC machines that collected dry blood spot samples and sent them to the hub for centralised testing, as well as urban and rural locations, and facilities serving high- and low-population areas. Severely ill people, including women with complications during pregnancy and delivery from these sites, were referred to the tertiary hospital for southern Malawi, Queen Elizabeth Central Hospital (QECH) [11]. The urban facility was 4 km from the tertiary facility and had a POC HIV testing machine, serving 143,515 people in 2023 [10,12]. The rural facility, 27 km away, served 10,089 and had no POC machine [10,12]. Both sites offered free services, including anti-retroviral therapy (ART). We compared EID uptake before and after the EEHS intervention in urban and rural facilities as a single unit of analysis, providing a disaggregated description for each.

### Study design

**Fig 1:**
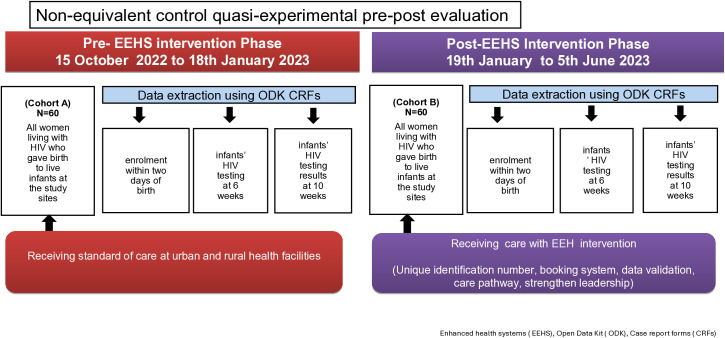
Pre-and post-EEHS intervention: non-equivalent control quasi-experimental design.

We conducted a non-equivalent control, quasi-experimental, pre-post evaluation [13] of the impact of an EEHS intervention on the enrolment of infants in the HIV care programme and HIV testing at six weeks in a real-world setting. Each phase, pre-intervention (15 October 2022–18 January 2023) and post-intervention (19 January to 5 June 2023), included a cohort of 60 women living with HIV who delivered live infants (Fig 1). The source population were all women who delivered at the participating facilities during the research period. We included all women living with HIV who gave birth to live infants at the study sites or whose infants were born before arriving (BBA), or live infants whose mothers had HIV but died after delivery. Overall, the study aimed to capture all infants exposed to HIV at birth at the study sites. Women with infants exposed to HIV were followed up to their 6-week appointment for early infant diagnosis services in both pre-and post-intervention cohorts at the study site.

### Pre- and post-intervention phase

The pre-intervention was the control period with standard care.

In the standard of care, the healthcare workers were responsible for identifying women with infants exposed to HIV when they accessed postnatal, immunisation, and family planning services by examining their passport books for documented HIV status or asking about it, in accordance with guidelines [14]. However, challenges arose in determining mothers’ HIV status and infants’ HIV exposure [5,8]. These included: i) healthcare workers avoided verifying clients’ HIV status due to concerns about stigma-related and unintended disclosure, especially since services were provided in crowded locations [5,8]; ii) there was inadequate documentation of HIV status [5,8]; iii) some healthcare workers focused on providing only one service and did not bother asking for HIV status [8]; iv) some clients changed their health passport book that had HIV status documentation to use one without the documentation [5,8].

The ART guidelines recommended that healthcare workers provide integrated services [14], but this was constrained by limited resources [5,6,8], including the POC machine’s capacity [5,8]. This limitation resulted in some infants being sent back without HIV testing at six weeks at the urban facility [5,6] ii) Healthcare workers failed to synchronise appointments and clustered their work shifts to extend their off-duty periods [5,8]. During the standard of care, Suwedi-Kapesa et al. found dysfunctional teams, misconduct, and limited supervision, which hindered healthcare worker coordination and accountability [5,8]. The guidelines recommended that healthcare workers provide EID services and identify high-risk infants exposed to HIV [14]; however, healthcare workers’ capacity was limited due to insufficient training [8]. Additional challenges in service provision within the standard of care are detailed by Suwedi-Kapesa et al.2025 [8].

**Fig 2:**
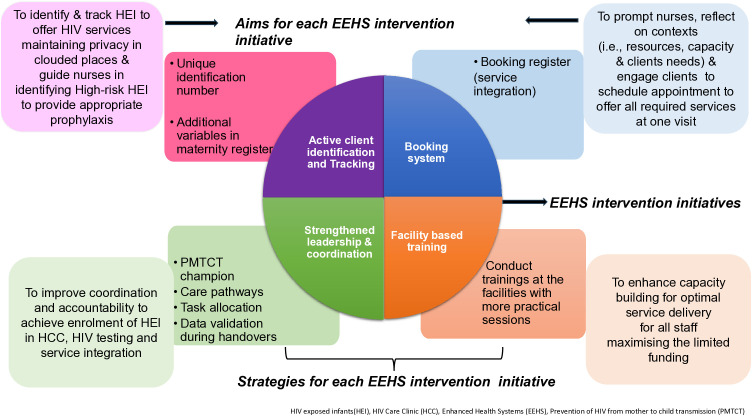
Implemented Context-appropriate co-designed EEHS intervention.

During post-intervention, women with infants exposed to HIV accessed services while healthcare workers implemented the co-designed EEHS intervention with four key components (Fig 2). The EEHS intervention has been described and published [8]. Below is the summary

**Active client identification**. Healthcare workers indicated and checked health passport books for unique identification numbers without directly asking clients about their HIV status in crowded spaces during daily service delivery [8]. Nurses utilised an additional page in the Maternity registers to identify infants with high-risk exposure [8]. The active identification aimed to identify and track women who were HIV positive and had infants exposed to HIV to enrol in HIV care clinics to receive appropriate EID services [8].

During clinic visits, all infants were weighed first by healthcare workers regardless of their HIV status. After weighing, the health passports books of mothers and infants were collected to verify HIV documentation [8]. Nurses then called each client for services in two common areas within the urban facility: a private room with adequate privacy and a larger, semi-private room with a screened bed, where other clients waited for services. They ensured HIV-positive clients or those without HIV documentation were cared for in a private room to protect the confidentiality of their status [8]. The same method was applied in rural areas to screen all clients, with nurses ensuring privacy and delivering personalised services within a single room. The nurses stressed the importance of testing, guiding women with unknown or outdated negative results to a trained counsellor for HIV testing and further services [8]. While waiting for HIV results for infants exposed to HIV using a point-of-care test, mothers and infants received services such as immunisation, family planning, and other essential care [8]. Once the results were available, ART and prophylaxis were administered.

Furthermore, maternity registers were updated with additional variables to prompt healthcare workers to identify infants exposed to HIV that were at high risk (S1 Text) [8]

### Booking system

This study used a dedicated booking system, Register (S2 Text), which incorporated variables prompting healthcare workers at both sites to consider their particular context and clients’ needs when scheduling appointments, thereby ensuring the provision of comprehensive, client-centred services [8]. These variables included clients’ preferences for continuing to access services at the facility after birth or obtaining a transfer to continue accessing six weeks care at a different facility of their choice, the days on which specific services are offered, the number of clients already booked on a given date relative to point-of-care (POC) capacity, and the actual appointment dates for each service [8]. This aimed to help healthcare workers set realistic dates for providing all services during a single visit, including HIV testing for infants exposed to HIV, postnatal care, family planning, immunisation, HIV, and ART services [8]. Nurses discussed all these necessary services with clients, recorded bookings at birth, and noted appointments at one week if the client delivered elsewhere. The booking nurse also corrected discrepancies, such as a mismatched appointment date at 6 weeks, and notified relevant providers [8]. At six weeks postnatal, nurses reviewed the register to identify expected clients and shared details of existing expert clients who had missed routine follow-up and tracing appointments.

**Strengthening leadership, data validation and care pathways.** Added a nurse midwife as a prevention of vertical transmission focal person to support the enrolment of infants exposed to HIV in care at birth, and complemented the EID focal person who monitored HIV testing from six weeks. Nurses validated data by verifying documents at handovers. Urban and rural facilities developed care pathways and prioritised and escorted clients to ensure they received all required services. Limited infrastructure prevented one-stop-shop services at the urban facility, while the rural facility offered some services in one room [8]. The urban facility escorted clients between service points to ensure all services were completed in a single appointment. The rural facility provided postnatal care, immunisation, and family planning within a single room due to its infrastructure; however, infant testing was conducted in a separate room, with clients being escorted. These efforts supported the booking system initiative, which aimed to enable clients to receive all services during a single visit [8].

**Facility-based training.** Before the intervention, healthcare workers received training and reflected on their context. The training was facility-based at the rural facility, and a nearby venue (a primary school hall) was used in the urban area due to limited space and a cholera outbreak. Registers and standard operating procedures were available at the study sites [8].

### Outcomes and definitions

The primary outcome was the proportion of infants exposed to HIV tested for HIV at six weeks. We defined HIV testing at six weeks as uptake of early infant diagnosis at 4–8 weeks of age [3,14]. The six-week testing outcome used the numerator as the total number of infants tested for HIV between 4 and 8 weeks, and the denominator was the total number of infants exposed to HIV born alive at or before arriving at the study site without documented transfer out or death outcomes. There were three secondary outcomes: (i) The proportion of infants exposed to HIV enrolled in HIV care programmes at birth was calculated by dividing the total number of infants enrolled in the HIV care programme at birth by the total number of infants exposed to HIV who were born alive at or before arriving at the study site (ii) the proportion of infants exposed to HIV enrolled at six weeks, the numerator was the total number of infants enrolled in HIV care programme at six weeks and the denominator was total number of infant exposed to HIV born alive at or born before arriving at the study site and (iii) the total number of infants identified as high-risk exposure with correct prophylaxis was the count of infants exposed to HIV who met the high-risk criteria and received appropriate prophylaxis, while the denominator was the total number of infants exposed to HIV and meeting the high-risk criteria exposure. We defined enrolment in an HIV care programme at birth as identifying and providing HIV care to the infants, documenting their exposed child patient card and documenting all details in the HIV care clinic register before post-natal discharge [14]. An infant at high-risk exposure was defined as an infant born from a woman with HIV who started antiretroviral therapy (ART) late (i.e., from seven months of pregnancy), or had never started ART, or started ART during labour or after birth or had interrupted ART during pregnancy or had a high viral load (> 1000 copies/ml) [14]. The correct high-risk prophylaxis was defined as 1.0 millilitres (mls) of nevirapine 24 hourly for infants that weighed less than 2500 grams(g), 1.5mls of nevirapine 24 hourly for infants that weighed less than 3000g to 2500g and ¼ tablet of paediatric Zidovudine, lamivudine and Nevirapine (2p) 24 hourly [14].

### Data collection

Trained researchers (LCSK & DN) prospectively collected routine data for women living with HIV and their infants using an electronic open data kit (ODK) case report form (CFR). They also took pictures of the same data from data sources without identifiers and entered this information into Excel to validate the electronically captured data. Data collection started on 15 October 2022 and continued until 18 January 2023 for the pre-intervention phase, and from 19 January to 5 June 2023 for the post-intervention phase. We extracted routine data directly from registers to maintain objectivity, thereby avoiding subjective data collection by researchers. We also considered care complete when documentation in routine care was completed [15,16]. The data sources were paper registers, the Malawi Ministry of Health (MoH) maternity register, the MoH HIV PCR sample logbook, the HIV care clinic card for infants exposed to HIV under 24 months and HIV care clinic registers. We added the MoH HIV testing service register on the data sources, following piloting and discussing with healthcare workers to identify HIV status for women without documented HIV results in the maternity registers. However, following the intervention, the Ministry of Health introduced a new version of HIV testing services registers (Version, Initial 1.11), which integrated rapid testing and counselling for HIV, syphilis, and hepatitis B. The new register had no provision to trace women from maternity registers who lacked HIV documentation status, and we discontinued using it.

We extracted demographic data for women and live infants at birth and study-related variables (S3 text). The prospective follow-up data were extracted from the data sources at three time points using three ODK CRFs: at birth for infant enrolment in HIV care, at six weeks for HIV testing data, and at ten weeks for test results from dried blood spot samples (DBS) with no outcome at the time of testing. We used various CRFs to ensure data quality, as forms could not remain incomplete for six to ten weeks. We merged the CRFs in R for complete participant information. Data was saved on the server and later uploaded in R 4.3.3 for cleaning, validation, and analysis.

### Sample Size calculation and Sampling

We calculated the sample size based on the comparison of two proportions between two independent groups, assuming equal sample sizes for the pre- and post-intervention periods. Secondary outcomes were not considered in the sample size estimation. The calculation was performed in **R** (version 4.3.3) using the power.prop.test() function from the **stats** package.

To determine the required sample size, we first specified the smallest clinically meaningful difference (effect size) to be detected. For proportions, the calculation required specifying $p_{\text{1}}$and $p_{\text{2}}$, representing the expected proportions at baseline and post-intervention, respectively. The absolute difference between these proportions ($\delta =p_{\text{2}}-p_{\text{1}}$) defines the effect size. Generally, smaller effect sizes require bigger sample sizes.

We explored various plausible values of $p_{\text{2}}$ within a range of expected post-intervention estimates. For the primary outcome of infants’ HIV testing at six weeks at the same health facility of birth, we assumed infant testing rate of 47% [5] in the pre-study period. The study power was set at 80%, representing the probability of detecting a true difference if one exists, and the significance level (α) was set at 0.05.

A standard formula for determining the sample size for each group was:


$$n=\text{2}{\left(\frac{Z_{\text{1}-\frac{\alpha }{\text{2}}}+Z_{\text{1}-\beta }}{\delta }\right)}^{\text{2}}$$

Where δ is the effect size α is the significance level, $Z_{1-\frac{\alpha }{2}}$ and $Z_{1-\beta }$ are critical values obtained from the standard normal distribution. The null hypothesis posited that there was no difference in the proportion of infants exposed to HIV tested at 6 weeks of age before and after implementing the EEHS intervention. Conversely, the alternative hypothesis suggested that a difference would exist. Sixty participants per study phase (pre- and post-intervention) provided 80% power to detect a 25% absolute difference in infant testing in the post-intervention period using a two-sided test with an alpha of 0.05. We used consecutive sampling to recruit all eligible participants meeting our criteria until we reached our target sample size.

### Statistical analysis

#### Handling missing data.

We initially assumed that the data were missing completely at random (MCAR). To evaluate this assumption, we examined the overall pattern of missingness, which indicated that approximately 3.4% of the data were missing. Although this low proportion suggested limited potential bias, we conducted formal tests to assess the validity of the MCAR assumption. A global test for MCAR was performed using Little’s test. In addition, chi-squared tests were used to examine associations between missingness indicators and observed categorical variables, with Fisher’s exact tests applied where expected cell counts were small. For continuous variables, we compared means between missing and observed groups using independent-sample *t*-tests.

To assess the robustness of our findings to potential violations of the MCAR assumption, we assumed a missing at random (MAR) mechanism and implemented multiple imputation by chained equations (MICE) as a sensitivity analysis. Results from complete case analyses were then compared with those obtained from the imputed datasets (S4 Text).

#### Descriptive statistics.

Our analysis followed the modified intention-to-treat principle. Infants with documented evidence of being transferred out from the study sites at birth or who died were excluded from the six-week HIV testing analysis. This is because clients have the right to choose where to receive their services after birth, and according to Malawi ART guidelines, clients who are known and confirmed to have transferred out or died are not classified as lost to follow-up (defaulter) [14]. In both phases, categorical variables were summarised as proportions (n/N), while continuous variables were summarised as median (interquartile range [IQR]). Associations between covariates and the study phase were explored using Chi-square or Fisher’s Exact test for categorical variables and using t-tests for continuous covariates.

#### Statistical modelling.

Unadjusted and adjusted logistic regression models were fitted to compute odds ratios (ORs) and 95% confidence intervals (CI). Statistical significance was defined as p < 0.05. In adjusted models, covariates were included if they had p < 0.1 in univariate analyses or established prior association with the outcome, such as the mother’s age, location of study [17–19] and mothers’ ART facility [5]. Model adequacy was assessed by examining multicollinearity using variance inflation factors (VIF); VIF = 1 shows no multicollinearity while high values, such as VIF > 5 indicate high collinearity. Goodness-of-fit was evaluated using the Hosmer-Lemeshow test, and influential observations were identified via leverage diagnostics. Residual patterns were assessed using deviance and Pearson residuals. All diagnostics were performed using the performance and DHARMa R packages. A sensitivity analysis evaluated the robustness of estimates to missing data, as detailed in the missing data section

All analyses were done using R (version 4.3.3).

## Results

The assessment of missing data revealed minor deviations from the MCAR assumption. Accordingly, a missing-at-random (MAR) mechanism was deemed more appropriate, and multiple imputation was employed to account for missingness in the multivariate regression analyses.

### Follow–up of participants

**Fig 3:**
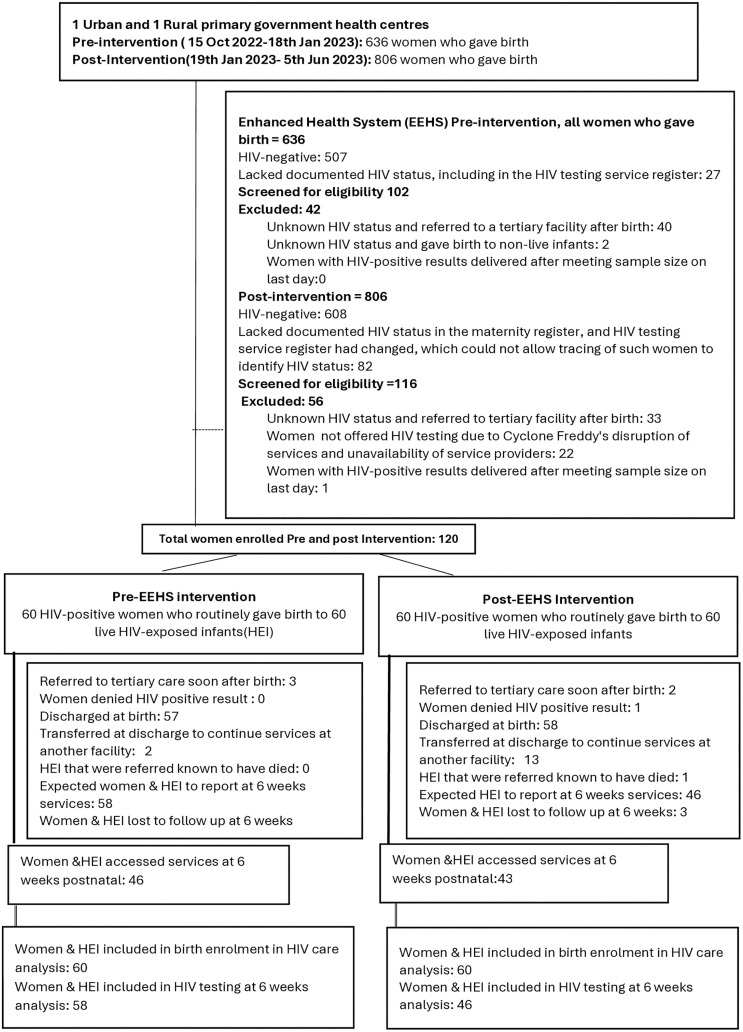
Follow up of extracted data for participants, pre-and post-intervention phases.

During the pre-intervention, 636 women gave birth at the study site. We confirmed that 60/636 (9.4%) of the women who delivered at the study site were eligible during our study period. Out of all women who delivered, there were 507/636 (78.9%) who were HIV negative and 27/636 (4.2%) lacked documented HIV status, including in the HIV testing service register, to determine their status. We excluded 42/102 (41.5%) because 40/102 (39%) were referred to the tertiary facility with unknown HIV status after giving birth, and 2/102 (0.02%) had unknown HIV status with non-live births (Fig 3).

Post-intervention, 806 women gave birth at the study site, and 60/806 (7.4%) were eligible. There were 608/806 (75%) women who were HIV negative, and 82/806 (10.2%) lacked documentation of HIV status and could not be traced. We excluded 56/116 (48.2%) because 22/116 (19%) were not offered HIV testing, 33/116 (28.4%) were referred to the tertiary facility with unknown HIV status after giving birth, and 0.9% (1/116) of women living with HIV delivered the night of the last day after we reached the sample size (Fig 3).

All women in each phase had live singleton births (Fig 3). One woman was in denial of her HIV positive result in the post-intervention cohort. Over the study period, five women and their infants were referred immediately after birth to tertiary hospitals due to complications such as post-partum haemorrhage and birth asphyxia. One referred infant died during the post-intervention phase, and four infants were alive and later continued accessing postnatal care at the primary delivery facility at six weeks. All women with uncomplicated births were discharged within 24 hours. Two women in the pre-intervention and 13 in the post-intervention were transferred from the delivery facility to their facilities for ARV access and subsequent health care services at discharge. Women with infants who remained in care at the delivery facility, 12/58, were lost to follow-up by six weeks in the pre-intervention and 3/46 in the post-intervention phase. Therefore, our analysis for infants exposed to HIV enrolment in the HIV care clinic at birth included all 60 women with live infants in the pre-and post-intervention. While the analysis of HIV testing at six weeks, for pre-and post-intervention, we included 58 and 46 infants, respectively, who remained in care (Fig 3).

### Characteristics of women living with HIV and had live infants, pre-and post-EEHS intervention at the Urban and Rural primary health facilities

Table 1 shows mothers of infants exposed to HIV characteristics for both intervention phases disaggregated for each study site. Mothers’ median age pre- and post-intervention was 27.5 (IQR, 23–31) and 28 (IQR, 23–32), respectively. Pre-intervention had 100% of women on ART, with 10% (6/10) initiating at 7 months of pregnancy or later. In contrast, post-intervention had 98% (59/60) of women on ART, with 13% (8/59) initiating at seven months of pregnancy and later. Of 60 mothers in the pre-intervention, 10 (16%) knew their viral load status *vs* 12 (16%) of 60 in the post-intervention phase. Among women with known viral load status at pre-intervention, 50% (5/10) had detectable levels and 25% (3/12) during the post-intervention.

**Table 1:** Characteristics of women living with HIV and live infants exposed to HIV pre-intervention and post-intervention phases.

	Pre-enhanced health system (EEHS) intervention			Post-EEHS intervention		
	Both N = N (%)	Urban N = N (%)	Rural N = N (%)	Both N = N (%)	Urban N = N (%)	Rural N = N (%)
Number of women with infants exposed to HIV	60/60 (100)	49/49 (100)	11/11 (100)	60/60 (100)	54/54 (100)	6/6(100)
***Age in years for women with infants exposed to HIV in categories***						
<24	23/60 (38)	16/49 (33)	7/11 (64)	17/60 (28)	15/54 (28)	2/6 (33)
25-34	27/60 (45)	25/49 (51)	2/11 (18)	34/60 (56)	31/54 (57)	3/6 (50)
>35	10/60 (17)	8/49 (16)	2/11 (18)	9/60 (15)	8/54 (15)	1/6 (17)
Median age (range)	27.5 (23 31.2)	28 (24 32)	23 (22 33.5)	28 (23 32)	28 (23,32)	26(24.5,28.2)
Women on Antiretroviral therapy (ART)	60/60 (100)	49/49 (100)	11/11 (100)	59/60 (98)	53/54 (98)	6/6 (100)
Accessing ART at delivery facility.	40/60 (66)	31/49 (63)	9/11 (81)	31/59 (52)	25/53 (47)	6/6 (100)
Women start ART ≥ 7 months of pregnancy	6/60 (10)	4/49 (8)	2/11 (18)	8/59 (13)	6/53 (11)	2/6 (33)
Ever stopped ART	1/60 (2)	1/49 (2)	0/11 (0)	2/59 (3)	2/53 (3)	0/6 (0)
Women with known Viral load	10/60 (16)	10/49 (20)	0/11 (0)	12/60 (20)	10/54 (19)	2/6 (33)
Women with detectable viral load	5/10 (50)	5/10 (50)	0/0 (0)	3/12 (25)	3/10(30)	0/2 (0)

Table 2 shows a descriptive summary of infants pre- and post-EEHS intervention. There was a balance of 60 infants exposed to HIV in both phases, with more infants born at the urban facility, 49 (82%) at pre-intervention and 54 (90%) at post-intervention *vs* 11 (18%) at the rural facility at pre-intervention and 6 (10%) at post-intervention. Pre-intervention had more infants at high-risk exposure, 13 (21%) *vs* 11 (18%). Of the 11 infants at high risk, 6 (55%) were correctly identified by healthcare workers in the pre-intervention period, versus 11 (85%) of 13 in the post-intervention period. In both phases, 16 (100%) infants were tested using DBS at the rural facility. Pre-intervention, 35(100%) infants were tested using a point-of-care machine at the urban facility. Post-intervention, 35(92%) were tested using a point-of-care machine, and 3(8%) used DBS due to a lack of cartilage. As Table 3 shows, there were no statistical differences in the characteristics of women with infants in the pre- and post-intervention phases.

**Table 2:** Descriptive summary for infants exposed to HIV Pre-and post- EEHS intervention at Urban and Rural Primary health facilities from birth to six weeks.

	Pre- enhanced health system (EEHS) intervention			Post-EEHS intervention		
	Both N= (%)	Urban N = N (%)	Rural N = N (%)	Both N = N (%)	Urban N = N (%)	Rural N = N (%)
All infants exposed to HIV born at the facility	60/60 (100)	49/49 (100)	11/11 (100)	60/60 (100)	54/54 (100)	6/6 (100)
Infants referred to the tertiary facility at birth	3/60 (5)	3/49 (8)	0/11 (0)	2/60 (3)	2/54 (4)	0/6 (0)
infants enrolled in HIV care clinic (HCC) at birth	47/60 (78)	37/49 (76)	10/11 (91)	55/60 (92)	49/54 (91)	6/6 (100)
**infants enrolled in HCC at birth, excluding those referred**	47/57 (82)	37/46 (80)	10/11 (90)	55/58 (95)	49/52 (94)	6/6 (100)
Infants ever enrolled in HCC	53/60 (88)	42/49 (86)	11/11 (100)	58/60 (97)	52/54 (96)	6/6 (100)
Clients refused HIV status & HCC enrolment	0/60 (0)	0/49 (0)	0/11 (0)	1/60 (2)	1/54 (2)	0/6 (0)
infant missed by health workers at birth HCC enrolment	10/57(18)	9/46(20)	1/11(9)	2/57(3)	2/51(3)	0/6 (100)
Infants at high-risk exposure	11/60 (18)	9/49 (18)	2/11 (18)	13/60 (21)	11/54 (20)	2/6 (33)
Correctly identified infants at high-risk	6/11 (55)	5/9 (55)	1/2 (50)	11/13 (85)	10/11 (90)	1/2 (50)
Infants at high risk with appropriate prophylaxis	2/11 (18)	1/9 (11)	1/2 (50)	10/13 (77)	9/11 (81)	1/2 (20)
Transferred out infants after HCC enrolment at birth	2/60 (3)	2/49 (4)	0/11(0)	13/60 (22)	12/54 (22)	1/6 (16)
Infants died before six months	0/60 (0)	0/49 (0)	0/11 (0)	1/60 (2)	1/54 (2)	0/6 (0)
Infants lost to follow-up before six months	12/60 (20)	12/49 (25)	0/11 (0)	3/60 (5)	3/54 (5)	0/6 (0)
Infants remained at the delivery site	58/60 (97)	47/49 (84)	11/11 (100)	46/60 (77)	41/54 (76)	5/6 (83)
	**Pre-EEHS intervention**			**Post-EEHS intervention**		
	Both N = N (%)	Urban N = N (%)	Rural N = N (%)	Both N = N (%)	Urban N= N (%)	Rural N= N (%)
**Primary Outcome: HIV-tested deoxyribonucleic acid polymerase chain reaction (DNA-PCR _) NB: excluding** **Transfer out and died)**	46/58 (79)	35/47 (75)	11/11 (100)	43/46 (93)	38/41 (93)	5/5 (100)
Median age for HIV testing (range)	6(6,6)	6(6,6)	6(6,6)	6(6,6)	6(6,6)	6(6,6)
HIV testing time Inter quartile range (IQR)	0	0	0	0	0	0
HIV-positive	0/46 (0)	0 (0)	0 (0)	2/43 (4)	2/38 (5)	0/5 (0)
Infants exposed without results	0/46 (0)	0/35 (0)	0/11 (0)	0/43 (0)	0/38 (0)	0 (0)
Infants alive at six weeks out of all infants	46/60 (76)	35/49 (74)	11/11 (100)	43/60 (72)	38/54 (70)	5/6 (83)
infants alive, not in HCC	0/60 (0)	0/49 (0)	0/11 (0)	1/60 (2)	1/54 (2)	0/6 (0)

**Table 3:** Univariable and Bivariate comparison between pre-and post-EEHS intervention.

Univariable analysis, stratified by pre-and post- enhanced health system (EEHS) intervention					
Variable	Level	Pre- EEHS,	Post-EEHS,	Odds ratio (OR) (95% CI)	P-value
Total participants (n)		**60**	**60**		
Mothers’ age (years) (mean (standard deviation (SD))		27.57 (5.68)	28.07 (6.26)		0.648
Study site (%)	Rural	11 (18.3)	6 (10.0)		0.295
	Urban	49 (81.7)	54 (90.0)		
Mothers’ Antiretroviral Therapy (ART) facility (%)	Outside delivery facility	20 (33.3)	29 (48.3)		0.137
	ART facility at the delivery facility	40 (66.7)	31 (51.7)		
Mothers ART duration (%)	Started ≥ 7 months of pregnancy	6 (10.0)	9 (15.0)		0.581
	Started < 7 months of pregnancy	54 (90.0)	51 (85.0)		
high-risk status (%)	At risk	49 (81.7)	47 (78.3)		0.819
	At high-risk	11 (18.3)	13 (21.7)		
**Bivariate analysis**					
HIV-tested Deoxyribonucleic acid polymerase chain reaction (DNA-PCR or point of care (POC) n (%)		46/58 (79)	43/46 (93)	3.74 (1.098 17.231)	0.052
Infants enrolled in HIV care clinic (HCC) at birth n (%)		47/60 (78)	55/60 (92)	3.04 (1.06 10.06)	0.048
Infants enrolled in HCC at birth excluding those referred (%)		47/57 (82)	55/58 (95)	3.90 (1.12 18.16)	0.048
infants ever enrolled in HCC n (%)		53/60 (88)	58/60 (97)	3.83 (0.88 26.48)	0.103
Infants at high risk with correct prophylaxis n (%)		2/11 (18)	10/13 (77)	15.00 (2.38. 145.89)	0.008
		46/58 (79)	43/46 (93)	3.74 (1.098 17.231)	0.052

### Primary and secondary outcomes

The primary outcome, among infants who remained at the facility and were alive at six weeks, the post-intervention group had a higher proportion of infants tested at six weeks, 93% (43/46), compared with 79% (46/58) in the pre-intervention (OR= 3.74, 95% CI; 1.10-17.23; p = 0.052). After adjusting for mothers’ age and ART facility and employing multiple imputation by chained equations to account for missing data, the evidence of improving infant testing at six weeks in post-intervention was stronger (OR = 4.36, 95% CI; 1.07-17.86, p = 0.040) (Table 4).

**Table 4:** Logistic regression model results for the association between EEHS intervention phases and infant enrolment in HIV care clinic at birth, adjusted for study site and mother characteristics. For the adjusted model, estimates were obtained using multiple imputation by chained equations (MICE) to account for missing data (n = 60 each phase).

Variable	Characteristic	Unadjusted Odds Ratio (OR)	95% CI		p-value	Adjusted OR	95% CI		p-value
Intervention phase	Pre-intervention	ref							
	Post-intervention	3.04	1.06	10.06	0.048	3.33	1.07	10.40	0.036
Maternal age	Yearly increase	0.99	0.91	1.07	0.753	0.98	0.90	1.08	0.712
Study site	rural	ref							
	Urban	0.32	0.02	1.72	0.279	0.25	0.03	2.16	0.205
Mother’s antiretroviral therapy(ART) facility	Outside delivery facility	ref							
	At delivery facility	0.91	0.31	2.50	0.856	0.93	0.31	2.76	0.896
Mother’s ART duration	Started ≥ 7 months of pregnancy	ref							
	Started < 7 months of pregnancy	0.86	0.13	3.50	0.847	NA	NA	NA	NA
Infants HIV high-risk exposure status	At risk	ref							
	At high-risk	0.85	0.27	3.26	0.798	NA	NA	NA	NA

The secondary outcome, the proportion of infants enrolled in HIV care clinics at birth in the post-intervention phase, was 92% (55/60) compared with 78% (47/60) in the pre-intervention phase (Table 2). In comparison with the pre-intervention phase, the odds of infant enrolment in an HIV care clinic at birth were significantly higher in the post-intervention phase: unadjusted OR 3.04 (95% CI 1.06-10.06, P = 0.048) (Table 3). In the analysis, adjusting for mothers’ age, study location, and the ART facility of the mother based on prior evidence study [5,17–19]. There was stronger evidence of effectiveness among infants exposed to HIV enrolment improvement in post-EEHS in the adjusted imputed model, with an adjusted OR (aOR) of 3.33 (95% CI 1.07-10.40, P = 0.036)

The post-intervention had a higher proportion of infants enrolled in the facility’s HIV care clinic by six weeks than the pre-intervention: 97% (58/60) vs. 88% (53/60); however, the observed difference was not statistically significant (OR = 3.83, 95% CI; 0.88-26.48; p = 0.103).

Among infants at a higher risk of HIV exposure, 77% (10/13) were given correct prophylaxis in the post-intervention phase compared with pre-intervention 18% (2/11). In comparison with pre-intervention, the odds of high-risk exposed infants given correct prophylaxis were significantly 15 times higher in post-intervention (95% CI 2.38-145.89; P = 0.008) (Table 3) (Table 5).

**Table 5:** Logistic regression model results for the association between EEHS intervention phases and infants testing at six weeks (primary outcome) adjusted for mothers’ characteristics. For the adjusted model, estimates were obtained using multiple imputation by chained equations (MICE) to account for missing data. (n = 58 pre-intervention and n = 46 post-intervention).

		Unadjusted			p-value	Adjusted			p-value
Variable	Characteristic	OR	95% CI			OR	95% CI		
Intervention phase	Pre-intervention	ref							
	Post-intervention	3.74	1.10	17.23	0.052	4.36	1.07	17.86	0.040
Maternal age (years)	Yearly increase	1.05	0.96	1.16	0.330	1.06	0.95	1.17	0.294
Mother’s antiretroviral therapy (ART) facility	Outside delivery facility	ref							
	At delivery facility	3.84	1.26	12.54	0.020	4.63	1.38	15.49	0.013
Mother’s ART duration	Started ≥7 months of pregnancy	ref							---
	Started < 7 months of pregnancy	0.38	0.02	2.15	0.372	NA	NA	NA	NA
Infants HIV high-risk exposure status	At risk	ref							
	At high-risk	2.01	0.50	13.50	0.383	NA	NA	NA	NA

In our sensitivity analysis, we found that the complete case analysis and multiple imputation yielded similar estimates. The only difference was observed in the confidence intervals, which were narrower in the imputation model compared to the complete case analyses (S4 Text).

## Discussion

Health system gaps limit EID service uptake, hindering progress toward a generation free of HIV by 2030 [2]. Understanding context and co-designed context-appropriate intervention is critical to improving EID services uptake. We compared HIV care enrolment at birth and testing at six weeks before and after implementing a context-appropriate EEHS intervention. We focused on urban and rural public primary facilities in Blantyre, Malawi, with and without POC machines. Enrolment of infants exposed to HIV at birth in HIV care programs improved from 78% pre-intervention, to 92% post-intervention. The odds of enrolment in HIV care at birth marginally increased in the intervention period, OR 3.04 (95% CI 1.06-10.06); p = 0.048, and in the adjusted imputed model, with an adjusted OR (aOR) of 3.33 (95% CI 1.07-10.40, P = 0.036). Similarly, we found improved proportion for HIV testing at six weeks from 79% pre-intervention, to 93% to post-intervention, OR 3.74 (95% CI 1.10-17.23; P = 0.052) with a statistically significant association for adjusted analysis with employed multiple imputation by chained equations to account for missing data (OR = 4.36, 95% CI; 1.07-17.86, p = 0.040) respectively. The unadjusted six-week HIV testing primary outcome showed borderline significance (p = 0.052). However, the effect size was substantial; the post-phase had 3.4 times higher odds of infants exposed to HIV testing at 6 weeks compared to before the intervention [20]. This suggests that the intervention may have clinical potential to improve testing at six weeks. Our findings show that the EEHS intervention may enhance the uptake of EID services, enrolment, and HIV testing at six weeks. Nevertheless, the precise size of this effect remains uncertain, and larger studies are needed to estimate its magnitude before accurate recommendations for broader implementation can be made.

The co-designed EEHS had multiple initiatives, with the booking system component aiming to improve service integration to enhance the uptake of EID. Although other studies [6,21–23]. have aimed to achieve service integration, the main difference is that earlier studies used pre-designed interventions [6,22,24,25]. In contrast, this study evaluated an intervention that was co-designed and developed with various stakeholders who possessed a thorough understanding and experience of the local context [9]. Whilst pre-designed interventions may initially reduce the need for up front resources for development [26]. However, their use might limit systems thinking and fail to address context related gaps/challenges. This could have unintended effects if interventions are ultimately unsuitable for providers, users, or settings [14,27].

Furthermore, service integration has varied definitions and implementations, with studies showing conflicting evidence of impact effectiveness [15] and lack of effect on EID service uptake [6,23,28].The WHO defines integrated health services as “health services that are managed and delivered so that people receive a continuum of health promotion, disease prevention, diagnosis, treatment, disease management, rehabilitation, and palliative care services, coordinated across different levels and locations of care based on their needs throughout their lifetime [29,30]. Our study defined patient-centred service integration as healthcare worker’s ability to provide all necessary services for mother and infant at a single appointment, regardless of using one or more rooms within the same health facility [8]. This definition, shaped by stakeholders, acknowledges the context, including limited infrastructure, healthcare worker availability, and the few daily tests the POC machine processes.

To facilitate service integration, previous studies have used a one-stop clinic model [6,22], while others have relied on referrals due to fragmented infrastructure in their settings [31]. Co-location is crucial for service integration [32]. Lilani et al. describe co-location as “different professional groups situated in the same workspace [32].” However, Lilani et al, did not specify that the workspace must be a single room or within the same health facility. Many studies define a one-stop clinic as one that provides services in a single room [6,24,25]. The Malawi 2022 clinical management of HIV guidelines [14], recommend HIV care to be integrated in the same clinic setting designated for clinical monitoring, preventive services and antiretroviral therapy integrated into the ART, Maternal and Neonatal Child Health clinics (MNCH) [14]. Besides co-location, other essential factors that support service integration include, co-management of information systems and good communication channels, such as clear responsibilities and care pathways [33]. Co-location is primarily impacted by limited or fragmented infrastructure, which often hinders the development of a genuine one-stop shop in LMICS within the same facility [5,6,24]. For example, previous studies in Malawi have highlighted challenges in integrating services for mothers and infants [5,6].

The formative evaluation of EID services in the same settings of this study indicated that healthcare workers made efforts and designated specific days to provide all services in one clinic room [5]. However, this had unintended outcomes. This caused clients with infants exposed to HIV to leave the hospital at six weeks without testing, as appointment numbers exceeded the POC machine’s capacity, with HIV testing at only 52% in 2018 [5]. A prior (2016) study in Malawi integrated an HIV/MNCH model with SMS. Some facilities offered a one-stop shop service, but retention at 12 months and infant HIV testing at six weeks did not improve [6]. While investing in co-location is crucial, it can require long-term investment [6,32]. To deliver integrated, person-centred care within a single setting with fragmented infrastructures, it is also crucial to address other key factors, such as communication channels [34]. These may necessitate short-term investments, as shown in this study, which aimed to improve care pathways and communication by implementing a booking system, data validation, and escorting, among other interventions that demonstrated potential to enhance EID outcomes.

Healthcare workers’ ability to identify infants at high risk of exposure and to provide correct prophylaxis improved after training, and new variables were added to maternity registers in the post-intervention phase. This finding supports the Malawi 2022 HIV management guidelines [14] and WHO recommendations [3] for managing infants at high-risk exposure. Likely, adding extra variables to the registers, as part of the client identification initiative, prompted healthcare workers to evaluate infants for high-risk exposure.

Our findings show that a co-designed context-appropriate intervention potentially enhances infants’ HIV care enrolment at birth and testing at six weeks, especially after adjusting for mothers’ age and ART facility. Other studies have suggested that maternal age, education level, parity, and location determine the uptake of EID [35]. In our study, the location was depicted as the study site, where one difference between urban and rural areas was the presence or absence of point-of-care machines. Although the urban facility mainly offered HIV testing to infants using POC machines; nevertheless, they also used DBS during post-intervention due to inconsistent availability of cartilages and did not want to return clients without HIV tests. We did not adjust for location or type of test in the HIV testing outcome because the final infant analysis numbers decreased due to transfers out at birth and deaths, compared to the birth analysis, which included all 60 infants.

Despite not reaching the 95% global EID testing target, HIV testing at six weeks with context-appropriate intervention rose to 93%, exceeding the 2023 Malawi national EID rate of 85% [2,4]. The different components of the EEHS likely contributed to improved outcomes. One of the key elements of this intervention was strengthening healthcare workers’ capacity through facility-based training to support other components of the intervention and EID service delivery. The Suwedi Kapesa et al 2024 and 2025 studies showed that healthcare workers had limited capacity to provide EID services in the same setting [5,8]. Building healthcare worker capacity likely led to improved service delivery.

Similarly, Suwedi Kapesa et al.‘s 2024 process evaluation of EID services in the same setting found that healthcare workers often failed to identify infants exposed to HIV because clients removed pages with HIV status from their books [5]. Additionally, there was a reluctance to ask clients about their HIV status in crowded places to protect privacy [5,9^9^]. The implementation of a unique identification number likely helped improve enrolment and HIV testing outcomes, as demonstrated in the co-designed enhanced health system intervention study by Suwedi Kapesa et al. (2025) [9]. This client identification aimed to enhance privacy, enable quick HIV status determination, and provide a more efficient method to establish HIV status even in busy environments, reducing the risk of missing infants exposed to HIV [9].

The appointment booking system helped healthcare workers reflect on the context and engage clients to schedule appointments [9]. We reported that infants lost to follow-up by 6 weeks were 12/58 before the intervention, compared to 3/46. The Malawi Clinical ART guidelines describe loss to follow-up as clients who have not come back to the clinic and are not known to have transferred out, stopped, or died [14]. This suggests that the booking system may have promoted patient-centred care and the provision of necessary services, including nurse-patient discussions and planning for subsequent care and access points [36].

Additionally, Suwedi Kapesa et al. (2024) found in their EID process evaluation that time was wasted due to poor coordination, and some services were not completed during appointments [5]. Based on the co-designed intervention, the care pathways, data validation, and escorting were intended to improve the coordination of services for infants exposed to HIV to access all necessary support, including HIV testing at six weeks [9]. The positive outcomes observed in this study suggest that this approach is likely to be effective. Escorting initiative is also similar to the findings of Ciamp et al. [37] on escorting clients between services when one-stop-shop options are unavailable, which were also found to be effective.

Based on improved outcomes at six weeks, this study suggests that the booking system, co-designed by Suwedi, Kapesa et al, likely achieved its aim, helping healthcare workers consider and synchronise appointment dates for mothers and infants, despite varying providers and fragmented infrastructure [9]. It improved healthcare workers’ ability to organise meaningful appointments, reflecting the capabilities of the point-of-care machine and the related needs of mothers and infants [9]. It provided practical guidance for healthcare workers to implement integrated services and use them as a tool to engage clients, which likely contributed to improvements in outcomes.

While the EEHS intervention centred on the health system, the prioritisation and clear care pathways initiatives present significant opportunities for leveraging male involvement to enhance service delivery. For instance, Window et al. [38] evaluated the impact of male participation on the uptake of EID. They found that prioritising women who brought their husbands to access HIV testing for infants exposed to HIV early significantly improved the uptake of EID at six weeks [38]

The co-designed intervention was evaluated in two facilities, both urban and rural, which is a strength of the study. Although the intervention was assessed at only two sites, its initiatives align with regional and national policies in Africa. For example, it supports the 2063 African Union Strategy [39], specifically aspiration 3, which promotes healthy and well-nourished citizens. Our findings demonstrated that the intervention could increase HIV testing rates at six weeks and help enrol infants in HIV care for early treatment, care, and support. Furthermore, it addressed health needs for mothers, including family planning and postnatal care, contributing to the goal of reducing preventable child and maternal health issues and deaths [39]. This study also aligns with the Sexual and Reproductive Health Rights (SRHR) in the SADC region 2019–2030 [40], which guides member states in providing integrated, equitable, and people-centred SRHR services [40]. Its objectives include reducing preventable maternal, newborn, child, and adolescent morbidity and mortality, preventing new HIV infections, and strengthening health systems to ensure universal access to SRHR. The study supports the multiple objectives of the SRHR strategy through (i) healthcare worker capacity building, (ii) active client identification, (iii) client-centred service integration, and (iv) Healthcare worker coordination and accountability, aiming to improve HIV detection at six weeks and enrolment in HIV care at birth [8]. Overall, this study aims to contribute to ending AIDS by 2030, emphasising strategic investments in advocacy and capacity building within the Catalytic Framework to end AIDS, TB, and eliminate malaria in Africa (2016–2030) [41].

In Malawi, the intervention also aligns with the country’s long-term strategic vision, Malawi 2063, which emphasises human capital development and highlights the importance of skilled, capable, and healthy human resources in reaching development goals [42]. This study suggests that facility-based training for healthcare workers likely promotes inclusiveness in human capital development. It involves various healthcare workers to increase capacity and reduce costs, which would otherwise limit training to fewer staff if centralised training were utilised, thereby strengthening the health workforce in line with the MW2063 vision goals [42]. The study advocates improving child and maternal survival and promoting future productivity by supporting a healthy population through systems thinking and a practical, comprehensive service-delivery approach [42]. This approach employs a booking system to provide a range of services for mothers and infants, despite resource constraints that often pose challenges. It also addresses the limitations in HIV testing and the enrolment of HIV-exposed infants into HIV care at birth. Since this study did not secure new resources, it offered practical strategies for achieving key outcomes with limited resources, thereby fostering resilience in accordance with the MW2063 vision [42]. Additionally, this study directly relates to three of the nine priority areas in Malawi’s Health Sector Strategic Plan III (HSSP III), 2023–2030: service delivery, human resources for health, and research [43]. Therefore, the initiatives evaluated in this study demonstrate strong alignment with both national and African regional policies, which is one factor that can aid scalability.

To scale up the intervention, it would be necessary to engage policymakers more actively, involving districts, communities, the private sector, regional and national stakeholders, and training institutions to adapt initiatives for client identification, service integration, capacity building, coordination, and accountability. Adapting strategies is necessary to address system challenges, rather than simply replicating them, given that contexts vary. This approach helps establish high-level, contextually appropriate practices and gather support [44]. This study did not perform a cost-effectiveness analysis due to limited resources. Given the short-term nature of the intervention, measuring the cost per Disability-Adjusted Life Year (DALY) averted would be difficult. Future research should explore whether a co-designed intervention comprising facility-based training, active client identification, a booking system, and coordination and accountability is cost-effective, which would be valuable for scaling up.

This study shows that interventions tailored to their specific context have the potential to be effective. Based on Suwedi Kapesa et al., the EEHS intervention was designed with consideration of its environment [9]. This intervention may not be directly transferable to other settings; however, the Suwedi Kapesa et al. intervention study [9], illustrated the process of designing an intervention suitable for the specific context. This study further demonstrates that context-appropriate interventions have the potential to be effective. Understanding the context is, therefore, key to increasing the chances of success in real-world applications. Our results suggest that in similar settings, a review of context with subsequent co-design of adaptations to service delivery, POC testing, and investment in integrated services may better achieve the WHO 2021 testing recommendations for IEH [3].

### Limitations of the study

We cannot draw causal inferences from our pilot findings because of the non-equivalent control quasi-experimental design, small sample size, and use of only two sites [13]. Our study used routinely collected data from real-world service implementation, which provided objectivity; however, it limited the variables we could examine. We adjusted findings based on the study site’s location (urban vs rural) for birth outcomes, which were critical to context and may explain participant characteristics like literacy, economic status, and education [18]. Routine data quality is often poor [45]. We enhanced it by prospectively collecting data, discussing gaps with healthcare workers, and triangulating data sources. For example, we identified gaps in the Maternity register regarding women’s HIV status, and healthcare workers advised researchers to utilise the HIV testing services register as the primary source for HIV information. However, when the register changed post-intervention, we used the same maternity register, which may have led to a higher number of women screened to reach the sample size than in the pre-intervention phase.

Selection bias may have occurred due to the use of consecutive sampling in the studied cohort and to the exclusion of severely ill patients transferred to the tertiary hospital from the study [15,16]. Ethically, we could not include seriously ill clients and deny them the care they needed at the tertiary hospital. Additionally, infants who died and were confirmed transferred were also omitted from the six-week analysis. According to Malawi ART guidelines, those who died and had confirmed transfers are not considered lost to follow-up. Therefore, the transferred-out patients were not followed up at their new health facilities to ascertain their outcomes at six weeks. Including outcomes at six weeks for the transferred-out clients who continued their care at new facilities that did not implement the intervention is also likely to introduce bias, and the outcomes may not reflect the intervention’s effectiveness [15,16].

Nevertheless, transferred-out outcomes were higher during the intervention. We believe that nurse-patient discussions, as part of the booking system, about a six-week appointment for care and subsequent access points, led more women to formally transfer to continue services at their preferred facilities. The increased number of infants transferring meant that six-week outcomes for all the women enrolled who delivered at the study facility could not be fully captured, which may affect the generalisability of our estimates.

To reduce measurement bias, we initially assessed the overall missing data pattern, finding about 3.4% was missing. Although this small proportion indicates a low risk of bias, we performed additional statistical tests to verify the MCAR assumption. We further examined how our results might be affected by a slight violation of the MCAR assumption; we performed a sensitivity analysis using multiple imputation by chained equations (MICE). Then, we compared the outcomes from the complete case analysis with those from the imputed datasets. For the enrolment and HIV model after imputation, we observed similar findings with slight changes in the CI, which were narrower compared to the adjusted, suggesting greater precision. Further details are available in the supplementary file. The adjusted analysis for the HIV model, including those transferred out and those who died, also showed similar findings; therefore, we maintained the exclusion of those who died and were transferred out to be consistent with the standard definition of loss to follow-up [14].

We consistently used the same data collectors throughout both the pre- and intervention phases [15,16]. The same tools, such as tablets running ODK software, were used for data collection. Routine data from registers were recorded to maintain objectivity and avoid subjective data collection by researchers [15,16]. To minimise confounding biases, we verified whether other interventions occurred at the study sites during the intervention that could explain the observed changes, but none were identified [16]. We also examined policy changes; during both the pre- and post-intervention periods, national policies for managing HIV-exposed infants remained unchanged [14]. The procedures for supply chain management of resources in HIV care remained consistent across both phases, and all resources utilised adhered to the existing framework [14]. However, during the intervention, a cholera outbreak and Cyclone Freddy occurred, potentially hindering the intervention due to service disruptions and increased demand to respond to these outbreaks. We further recognise that, although we assessed various circular changes, others may have been missed [16].

## Conclusion

In conclusion, we found that an EEHS intervention with active client identification, client-centred service integration, healthcare worker coordination and accountability and increased capacity for healthcare worker to provide EID services could increase uptake of infants exposed to HIV enrolment at birth and HIV testing at six weeks. This study shows that interventions tailored to their specific context have the potential to be effective. Further studies should evaluate this EEHS intervention using a cluster-randomised controlled trial approach. In addition, the fidelity and acceptability of this EEHS intervention should be assessed to continue adapting implementation in line with context.

## Supporting information

S1 Checklist Inclusivity in global research questionnaire. (DOCX)

S1 Text Additional page of variables for register. (PDF)

S2 Text Booking system Register. (PDF)

S3 Text Data extraction from. (PDF)

S4 Text Sensitivity data analysis, additional results. (PDF)

## References

[1] Joint United Nations Programme on HIV/AIDS. Transforming Vision into Reality: The 2024 Global Alliance Progress Report on Ending AIDS in children by 2030. https://www.unaids.org/en/resources/documents/2024/transforming-vision-into-reality. Accessed 2024 October 8.

[2] Joint United Nations Programme on HIV/AIDS. The urgency of now: AIDS at a crossroads. 2024. https://www.unaids.org/sites/default/files/media_asset/2024-unaids-global-aids-update_en.pdf

[3] World Health Organisation. Guidelines: updated recommendations on HIV prevention, infant diagnosis, antiretroviral initiation and monitoring. 2021. https://www.who.int/publications/i/item/9789240022232 33822559

[4] Joint United Nations Programme on HIV/AIDS. Global data on HIV epidemiology and response: Malawi 2023 HIV and AIDS Estimates. 2024. https://aidsinfo.unaids.org/

[5] Suwedi-Kapesa LC, Nyondo-Mipando AL, Choko A, Obasi A, MacPherson P, Desmond N. Process Evaluation of Services for HIV-Infected Post-Partum Women and HIV-Exposed Infants in Primary Health Care Blantyre Malawi. Health Serv Insights. 2024;17:11786329231224623. doi: 10.1177/11786329231224623 38322594 PMC10846036

[6] Mwapasa V, Joseph J, Tchereni T, Jousset A, Gunda A. Impact of Mother-Infant Pair Clinics and Short-Text Messaging Service (SMS) Reminders on Retention of HIV-Infected Women and HIV-Exposed Infants in eMTCT Care in Malawi: A Cluster Randomized Trial. J Acquir Immune Defic Syndr. 2017;75 Suppl 2:S123–31. doi: 10.1097/QAI.0000000000001340 28498181

[7] Wight D, Wimbush E, Jepson R, Doi L. Six steps in quality intervention development (6SQuID). J Epidemiol Community Health. 2016;70(5):520–5. doi: 10.1136/jech-2015-205952 26573236 PMC4853546

[8] Ministry of Health M. Malawi population-based HIV impact assessment 2020-2021. 2022. https://dms.hiv.health.gov.mw/link/wq29x5kc

[9] Suwedi-Kapesa LC, Choko AT, Nyondo-Mipando AL, Zimba JH, Lipipa E, Nothale D, et al. Developing an intervention to improve early infant HIV diagnosis service uptake among postpartum women in Malawi’s primary healthcare using a co-designing approach with stakeholders. PLOS Glob Public Health. 2025;5(4):e0004426. doi: 10.1371/journal.pgph.0004426 40261865 PMC12013899

[10] Government of Malawi Ministry of Health and Population. EID- & VL strategic and implementation plan. 2022.

[11] Government of the Republic of Malawi Ministry of Health. National community health strategy 2017-2022. Lilongwe: Ministry of Health. 2017. https://www.healthynewbornnetwork.org/hnn-content/uploads/National_Community_Health_Strategy_2017-2022-FINAL.pdf

[12] National statistical office. Malawi Population and Housing Census Population Projections 2018-2050. 2018. https://malawi.unfpa.org/sites/default/files/resource-pdf/2018%20Malawi%20Population%20and%20Housing%20Census%20Main%20Report%20%281%29.pdf

[13] McDavid JC, Huse I, Hawthorn LR. Program evaluation and performance measurement: An introduction to practice. Sage Publications. 2018.

[14] Ministry of Health and Population. Clinical management of HIV in children and adults. 2022. https://www.differentiatedservicedelivery.org/wp-content/uploads/Malawi-Clinical-HIV-Guidelines-2022-edition-5.pdf

[15] Handley MA, Lyles CR, McCulloch C, Cattamanchi A. Selecting and Improving Quasi-Experimental Designs in Effectiveness and Implementation Research. Annu Rev Public Health. 2018;39:5–25. doi: 10.1146/annurev-publhealth-040617-014128 29328873 PMC8011057

[16] Harris AD, McGregor JC, Perencevich EN, Furuno JP, Zhu J, Peterson DE, et al. The use and interpretation of quasi-experimental studies in medical informatics. J Am Med Inform Assoc. 2006;13(1):16–23. doi: 10.1197/jamia.M1749 16221933 PMC1380192

[17] Mbeya Munkhondya TE, Smyth RM, Lavender T. Facilitators and barriers to retention in care under universal antiretroviral therapy (Option B+) for the Prevention of Mother to Child Transmission of HIV (PMTCT): A narrative review. International Journal of Africa Nursing Sciences. 2021;15:100372. doi: 10.1016/j.ijans.2021.100372

[18] Astawesegn FH, Mannan H, Stulz V, Conroy E. Understanding the uptake and determinants of prevention of mother-to-child transmission of HIV services in East Africa: Mixed methods systematic review and meta-analysis. PLoS One. 2024;19(4):e0300606. doi: 10.1371/journal.pone.0300606 38635647 PMC11025786

[19] Fassinou LC, Songwa Nkeunang D, Delvaux T, Nagot N, Kirakoya-Samadoulougou F. Adherence to option B + antiretroviral therapy and associated factors in pregnant and breastfeeding women in Sub-Saharan Africa: a systematic review and meta-analysis. BMC Public Health. 2024;24(1):94. doi: 10.1186/s12889-023-17004-9 38183014 PMC10768427

[20] Sullivan GM, Feinn R. Using Effect Size-or Why the P Value Is Not Enough. J Grad Med Educ. 2012;4(3):279–82. doi: 10.4300/JGME-D-12-00156.1 23997866 PMC3444174

[21] Kiragu K, Collins L, Von Zinkernagel D, Mushavi A. Integrating PMTCT into maternal, newborn, and child health and related services: experiences from the global plan priority countries. J Acquir Immune Defic Syndr. 2017;75(Suppl 1):S36–42. doi: 10.1097/QAI.0000000000001323 28398995

[22] Geelhoed DLY. Integrated maternal and child health services in Mozambique: Structural health system limitations overshadow its effect on follow-up of HIV-exposed infants. BMC Health Serv Res. 2013;13(207):1–8. doi: 10.1186/1472-6963-13-207 23758816 PMC3679935

[23] Prescott M, Boeke C, Gotora T, Mafaune HW, Motsi W, Graves J. Integration of EPI and pediatric HIV services for improved ART initiation in Zimbabwe. 2017;1. 10.7910/DVN/HIXN40

[24] Washington S, Owuor K, Turan JM, Steinfeld RL, Onono M, Shade SB, et al. Implementation and operational research: effect of integration of HIV care and treatment into antenatal care clinics on mother-to-child HIV transmission and maternal outcomes in Nyanza, Kenya: results from the SHAIP cluster randomized controlled trial. J Acquir Immune Defic Syndr. 2015;69(5):e164-71. doi: 10.1097/QAI.0000000000000656 25886930 PMC4837126

[25] Gamell A, Luwanda LB, Kalinjuma AV, Samson L, Ntamatungiro AJ, Weisser M, et al. Prevention of mother-to-child transmission of HIV Option B+ cascade in rural Tanzania: The One Stop Clinic model. PLoS One. 2017;12(7):e0181096. doi: 10.1371/journal.pone.0181096 28704472 PMC5507522

[26] World Health Organisation. Consolidated guidelines on HIV prevention, testing, treatment, service delivery and monitoring: recommendations for a public health approach. World Health Organisation. 2021. https://www.who.int/publications/i/item/9789240031593 34370423

[27] De Savigny D, Adam T. Systems thinking for health systems strengthening. World Health Organization. 2009.

[28] Okusanya B, Kimaru LJ, Mantina N, Gerald LB, Pettygrove S, Taren D, et al. Interventions to increase early infant diagnosis of HIV infection: A systematic review and meta-analysis. PLoS One. 2022;17(2):e0258863. doi: 10.1371/journal.pone.0258863 35213579 PMC8880648

[29] World Health Organisation. Framework on integrated, people-centred health services. 2016. https://apps.who.int/gb/ebwha/pdf_files/wha69/a69_39-en.pdf

[30] World Health Organisation. Continuity and coordination of care: a practice brief to support implementation of the WHO Framework on integrated people-centred health services. 2018. https://www.who.int/publications/i/item/9789241514033

[31] Wiegert K, Dinh T-H, Mushavi A, Mugurungi O, Kilmarx PH. Integration of prevention of mother-to-child transmission of HIV (PMTCT) postpartum services with other HIV care and treatment services within the maternal and child health setting in Zimbabwe, 2012. PLoS One. 2014;9(6):e98236. doi: 10.1371/journal.pone.0098236 24915422 PMC4051591

[32] Lalani M, Marshall M. Co-location, an enabler for service integration? Lessons from an evaluation of integrated community care teams in East London. Health Soc Care Community. 2022;30(2):e388–96. doi: 10.1111/hsc.13211 33152144 PMC9290730

[33] Liapi F, Chater AM, Randhawa G, Stephenson A, Pappas Y. Factors that influence inter-organisational integration: a qualitative exploration of service providers’ perspectives from an integrated care initiative. BMC Health Serv Res. 2025;25(1):947. doi: 10.1186/s12913-025-13051-7 40640843 PMC12247228

[34] Valentijn PP, Schepman SM, Opheij W, Bruijnzeels MA. Understanding integrated care: a comprehensive conceptual framework based on the integrative functions of primary care. Int J Integr Care. 2013;13:e010. doi: 10.5334/ijic.886 23687482 PMC3653278

[35] Makau G, Okwara F, Oyore J. Determinants of early infant diagnosis and treatment of HIV among exposed infants in informal settlements in Nairobi, Kenya. East Cent Afr Med J. 2015;2:74–9.

[36] World Health Organisation. Consolidated guidelines on person-centred HIV strategic information: strengthening routine data for impact. World Health Organization. 2022. https://www.who.int/publications/i/item/9789240055315

[37] Ciampa PJ, Burlison JR, Blevins M, Sidat M, Moon TD, Rothman RL, et al. Improving retention in the early infant diagnosis of HIV program in rural Mozambique by better service integration. J Acquir Immune Defic Syndr. 2011;58(1):115–9. doi: 10.1097/QAI.0b013e31822149bf 21546845

[38] Window M, Nyondo-Mipando AL, Kalanga N. Male involvement enhances the uptake of early infant diagnosis of HIV services in Thyolo, Malawi: A non-equivalent control group quasi-experimental study. PLoS One. 2023;18(2):e0281105. doi: 10.1371/journal.pone.0281105 36812286 PMC9946214

[39] DeGhetto K, Gray JR, Kiggundu MN. The African Union’s Agenda 2063: Aspirations, challenges, and opportunities for management research. Africa Journal of Management. 2016;2(1):93–116.

[40] Southern African Development Community (SADC) Secretariat. Score Card for Sexual and Reproductive Health and Rights in the SADC Region Fast Tracking the Strategy for SRHR in the SADC Region 2019 – 2030. 2019. https://healtheducationresources.unesco.org/library/documents/score-card-sexual-and-reproductive-health-and-rights-sadc-region-fast-tracking

[41] Union A. Catalytic framework to end AIDS, TB and eliminate malaria in Africa by 2030. 2016. https://au.int/sites/default/files/newsevents/workingdocuments/27513-wd-sa16949_e_catalytic_framework.pdf

[42] Government of Malawi. Malawi’s Vision an inclusively wealthy and self-reliant Nation 2063. Lilongwe, Malawi: National Planning Commission. 2020. https://malawi.un.org/sites/default/files/2021-01/MW2063-%20Malawi%20Vision%202063%20Document.pdf

[43] Government of Malawi. Health Sector Strategic Plan III 2023-2030, Reforming for Universal Health Coverage 2. 2023. https://www.prepwatch.org/wp-content/uploads/2024/07/HSSP-III-Print-Version-2023-01-09_v1_Cleaned.pdf

[44] Milat AJ, King L, Bauman AE, Redman S. The concept of scalability: increasing the scale and potential adoption of health promotion interventions into policy and practice. Health Promot Int. 2013;28(3):285–98. doi: 10.1093/heapro/dar097 22241853

[45] O’Hagan R, Marx MA, Finnegan KE, Naphini P, Ng’ambi K, Laija K, et al. National assessment of data quality and associated systems-level factors in Malawi. Glob Health Sci Pract. 2017;5(3):367–81. doi: 10.9745/GHSP-D-17-00177 28963173 PMC5620335

