# Supplementary material for: A context-responsive health systems intervention improves the uptake of early infant HIV diagnosis: Controlled before and after study in Malawi
Source: PLOS Glob Public Health. 2026 Apr 21;6(4):e0006269. doi: 10.1371/journal.pgph.0006269 (PMC13099088; doi:10.1371/journal.pgph.0006269)
Supplement: S1 Checklist — (DOCX) [file pgph.0006269.s001.docx]

Inclusivity in global research

PLOS’ policy on inclusivity in global research aims to improve transparency in the reporting of research performed outside of researchers’ own country or community and ensures that PLOS publications reporting global research adhere to high standards for research ethics and authorship. Authors of relevant research articles may be asked to complete the questionnaire below, which outlines ethical, cultural, and scientific considerations specific to inclusivity in global research. This questionnaire may be requested when researchers have travelled to a different country to conduct research, if research uses samples collected in another country, research with Indigenous populations or their lands, or if research is on cultural artefacts. Researchers travelling to another country solely to use laboratory equipment will not normally be required to complete the questionnaire. However, the questionnaire can be requested at the journal’s discretion for any submission – if you have been requested to complete this questionnaire by the PLOS journal you submitted to, please do so.

Please complete the questionnaire below and include this as a Supporting Information file with your manuscript. Note that if your paper is accepted for publication, this checklist will be published with your article in the supporting information files. Please ensure that you reference the checklist in the main body of your manuscript. We suggest adding a subsection ‘Inclusivity in global research’ to your Methods section and adding the following sentence: “Additional information regarding the ethical, cultural, and scientific considerations specific to inclusivity in global research is included in the Supporting Information (SX Checklist)”

The questions have been designed to be applicable to a wide range of study types, and there are subsections for both human subjects research and non-human subjects’ research. If any of the questions are not relevant to your research, please mark them as “N/A” as appropriate.

**Ethical considerations, permits and authorship**

*This section is applicable to all research types.*

Provide details as to who granted permissions and/or consent for the study to take place in the Methods section of your manuscript. This should include the names of **all** ethics boards, governmental organizations, community leaders or other bodies that provided approval for the study. If individuals provided approval refer to these people by their role or title but do not list their name(s).

Reported on page number: Page 7

1. College of Medicine Research & Ethics Committee (P.11/23-0438) and the Liverpool School of Tropical Medicine Research Ethics Committee (22-025).

2. Blantyre district health management and research committee

3. Director of Health and Social Services in Blantyre district

4. Respective Health facilities management team members and representatives from the community for the health advisory committee

If there were any deviations from the study protocol after approval was obtained, please provide details of these changes in the Methods section of your manuscript.
Did this study involve local collaborators that are residents of the country where the research was conducted, or members of the community studied? If you do not have any authors from said communities, please provide an explanation for this below.

No

Reported on page number: N/A

1. Yes, the study involved collaborators from the country (Augustine Choko ^1,2^, James Chirombo^1,2^ and another co-authors were part of the Blantyre district health office involved in the study ( Dorcus Nothale^4^)

2. The principal researcher is from the country

Everyone listed as an author should meet PLOS’ criteria for authorship and all individuals who meet these criteria should be included in the author byline, rather than the acknowledgements. For further information please see the journal’s Authorship Policy. (Yes, they do)

**Human subjects research (e.g. health research, medical research, cross-cultural psychology)**

Did you obtain written informed consent from a representative of the local community or region before the research took place? How did you establish who speaks for the community? Details of written informed consent obtained from study participants should be reported separately in the Methods section of your manuscript.

1. Before the study, we made a research presentation to the Blantyre district health management team and research committee, and we later received approval to conduct the study.
2. We made a presentation to the primary facilities, including the management teams for the facilities, who also invited community representatives who speak on behalf of the community as members of health advisory committees

How did members of the local community provide input on the aims of the research investigation, its methodology, and its anticipated outcome(s)?

This phase of evaluating an intervention was built on from an initial research work where we co-designed the intervention with different key stakeholders which embraced a lot of engagement from the stakeholders.

Following the co-design phase of the intervention, we conducted workshops with all staff at the study sites and offered additional training. The training integrated feedback from staff regarding the feasibility of the intervention and their reflections on how well it could be embraced by everyone in the setting. This process built on the initial co-design, engaging all implementers within the facilities.

Following this study, interviews were conducted with healthcare workers and clients to gather their inputs, and the findings are drafted for another publication.

When engaging with the local community, how did you ensure that the informed consent documents and other materials could be understood by local stakeholders?

In preparation for intervention implementation, healthcare workers were invited to participate in the training in writing and had the option to opt in or not. Regarding data extraction, we did not engage directly with patients; instead, we collected routine data to ensure objectivity. The ethics committees approved this process, and the director of health and social services, along with the research team, authorised the study.

In addition, we had engagement with management, staff, and community representatives who are part of the hospital advisory committee to discuss the study and obtain their input before starting the actual data collection.

Will the findings of the research be made available in an understandable format to stakeholders in the community where the study was conducted (e.g. via a presentation, summary report, copies of publications, etc.)? Please provide details of how this will be achieved.

Yes, for example, the first and second phases that informed the intervention evaluated and reported in this study had their findings summarised and validated during the training of healthcare workers at two study sites. Overall findings will also be disseminated at the district health office, engaging the NGO and key healthcare workers from the facilities, and subsequently at primary facilities involving community representatives.

**Non-human subjects research using specimens/ animals collected as part of the study, or those housed in archival collections. Examples include archaeology, paleontology, botany and zoology.**

Did the permission you obtained from a local authority to perform the study include an agreement on access to outputs and benefit sharing? This may include procedures to enable fair distribution of the benefits and resources arising from the research performed. Please include any details of Prior Informed Consent and Benefit Sharing Agreements obtained. These may be required by field-specific regulations, for example the Convention on Biological Diversity (CBD) and the associated Nagoya Protocol.

N/A

If the material used in your study was imported, please A) provide the year it was imported and B) indicate whether permits were obtained to import/export the materials used, C) provide details of any permits obtained. If this information is not available, please indicate this.

N/A

If you used archival specimens, please state how the material used in your study was acquired by the institute it is held in and provide details of any permits obtained for the original excavations/ sample collection. If this information is not available, please indicate this.

N/A

How was the potential cultural significance of the materials collected in your study to local communities considered in your research design? Were Indigenous peoples and/or local researchers and institutions involved with archaeological excavations / collection of specimens? If so, please provide a description of their involvement.

N/A

If your manuscript includes photographs of human remains, please indicate whether authors obtained permission from descendants or affiliated cultural communities to do so.

No
