# Supplementary material for: A context-responsive health systems intervention improves the uptake of early infant HIV diagnosis: Controlled before and after study in Malawi
Source: PLOS Glob Public Health. 2026 Apr 21;6(4):e0006269. doi: 10.1371/journal.pgph.0006269 (PMC13099088; doi:10.1371/journal.pgph.0006269)
Supplement: S1 Text — (PDF) [file pgph.0006269.s002.pdf]

Additional Variables in maternity register

Continuation of Baby								
**Continuation of PMTCT management**								
	VL result		Adherent		HEI Risk status		2P	
	Yes	No	Yes	No	High	Low	70	
	67		68		69			
	H	LDL	Y	N	H	L		
	H	LDL	Y	N	H	L		
	H	LDL	Y	N	H	L		
	H	LDL	Y	N	H	L		
	H	LDL	Y	N	H	L		
Page Summary								

Field Number

Note: This part is completed to support healthcare workers

Numbers 67, 68, and 69 stand for variables, viral load result, adherence and prophylaxis respectively. The numbers were used to pilot how the rest of variables are entered on DHIS 2 (Reporting platform) for reporting. However, these variables were not reported on DHIS 2 because this was a pilot.

The letters have different meanings, as explained below. HCW circles one letter in each column depending on their assessment or action.

Viral load result column, H mean high viral load result, and LDL mean low level detected

Adherent column Y mean yes, and N mean No to the mother of HIV exposed infant being adherent to ARVs

The high-risk status column H means the infant is at high risk, and L infant is at low risk

2P is a high-risk prophylaxis given
