# Supplementary material for: A context-responsive health systems intervention improves the uptake of early infant HIV diagnosis: Controlled before and after study in Malawi
Source: PLOS Glob Public Health. 2026 Apr 21;6(4):e0006269. doi: 10.1371/journal.pgph.0006269 (PMC13099088; doi:10.1371/journal.pgph.0006269)
Supplement: S3 Text — (PDF) [file pgph.0006269.s004.pdf]

Data extraction from

Data extraction form of HEIs enrolment and HIV ascertainment of HEIs at 6 weeks of age, in selected health facilities Before and After EEHs intervention

Date of data collection………. Data collected for the month of……………… Study site…………………

Data collected by: ………………..Signature………………… Or ………….: Signature …………………

Or Researcher: Leticia Suwedi-Kapesa Signature…………………..

Maternity Register									HEI under 24 months(pink card)or logbook if results are not on the card							
Month	Serial #	facility code	ART status	ART #	ARTFac	Mage	Infant surv	HEI DOB	HEIHCC	RES	Heidoenrr	HEI test	Test T WKS	Test typ	Res	6Wks out

Key:

Facility code: a number assigned to the study site

Month: month of data collection

ART status: Whether mother is on antiretroviral therapy or not? Indicate yes or no

ART #: if mother is on ART, whats the ART number

ART FAC: The facility where mother collects ART

Mage: Mothers age

Infant surv: Is the the infant alive or not at birth indicate yes

HEIDOB: HIV-exposed infant date of birth

HEIHCC: is the HIV exposed infant enrolled in HIV care or not? Indicate yes or no

Res: reason for no enrolment in HIV care i.e died, transferred out, no reason

Heidoenrr: HIV-exposed infant date of enrolment in HIV care clinic

HEItest: Whether the infant has received an HIV test aimed for 6 weeks or not

Test TWKS: Testing time in weeks (Indicate the age of the infant at the time of doing testing in weeks

Test typ: Test type, what type of test has been offered to the HEI? Is it dry blood spot sample or Point of care?

Res: *Result for the test, is it positive or negative?*

6WKsout: What is the outcome of the HEI at 6 weeks? (died, alive in care, transferred out, lost to follow up)

Comments/ notes …….

……..

Total number of HEIs at 2 months in a cohort…………… Total number tested for DNA PCR ……………..
