# Supplementary material for: A context-responsive health systems intervention improves the uptake of early infant HIV diagnosis: Controlled before and after study in Malawi
Source: PLOS Glob Public Health. 2026 Apr 21;6(4):e0006269. doi: 10.1371/journal.pgph.0006269 (PMC13099088; doi:10.1371/journal.pgph.0006269)
Supplement: S4 Text — (PDF) [file pgph.0006269.s005.pdf]

Supplementary materials

Missing data handling

We first summarised the data to obtain the overall rate of missingness. We found a missing rate of 3.4%. The 3 variables with missing data include hiv status (13%), enrolment at time 2 at 14% and binary_test_hei at 27%.

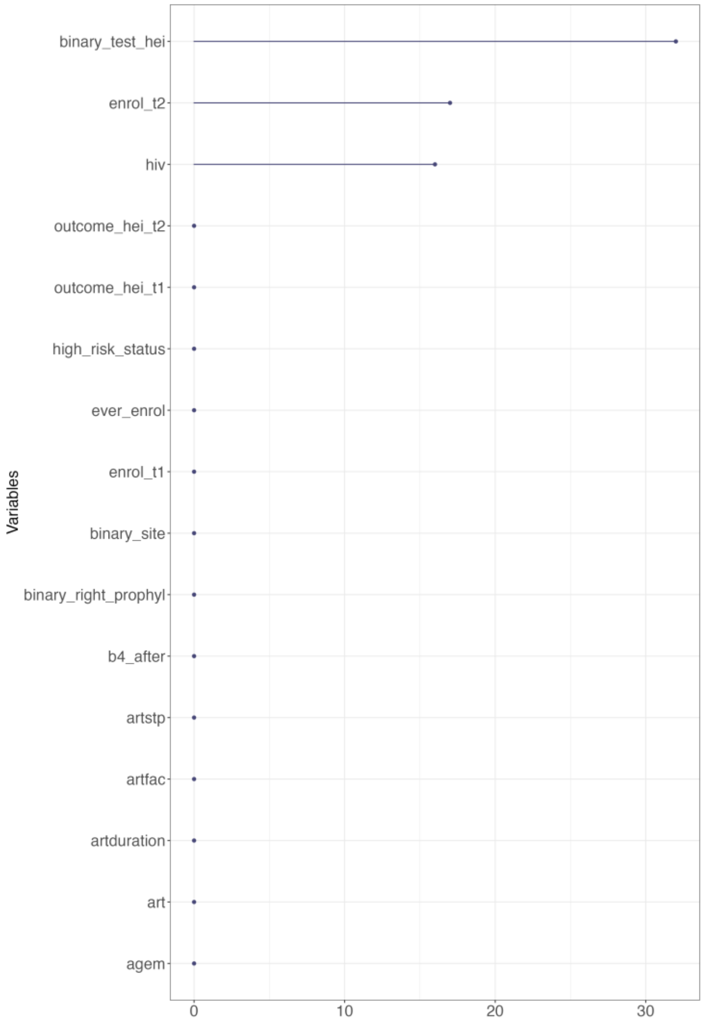

We assumed that the data were missing completely at random (MCAR). Firstly, we performed Little's MCAR test on the data which returned a significant result indicating that MCAR was violated. We then conducted statistical tests to check for association between missingness indicators and observed categorical variables using Chi-squared tests (Fisher's exact tests were used where expected cell counts were small). continuous variables, means between missing and observed groups were compared using independent sample t-tests.

Assessment of variable-level Fisher's exact tests indicated that the majority of variables were consistent with the MCAR assumption, although a few showed evidence of systematic missingness. This observation was also supported by the logistic regression

approach to estimate the probability of missingness as a function of observed variables and computed odds ratios to identify predictors of missingness. We observed a few variables with significant ORs thus violating the MCAR assumption. Given that perfect MCAR is rare in observational data, and that the observed violations were limited, we proceeded under the Missing at Random (MAR) assumption. To evaluate the robustness of our findings to different missing data handling strategies, we conducted a sensitivity analysis comparing results from complete case analysis (case deletion) with those obtained using multiple imputation. We applied this sensitivity to the adjusted models.

In the both models, with enrolment time and hiv as the outcome variable, the imputation and complete case analysis models provided identical results. The only difference was on the confidence intervals which were relatively narrower compared to the complete case analysis.

Table 1: Comparison of complete case analysis and multiple imputation for the enrolment model

Term	MICE estimate	CI	GLM estimate	CI
Intervention phase				
Pre-intervention	Ref			
Post-intervention	3.33	1.07 – 10.4	3.33	1.34 – 11.3
Maternal age	0.98	0.90 – 1.08	0.98	0.89 – 1.08
Study site				
Rural	Ref			
Urban	0.25	0.03 – 2.16	0.25	0.01 – 1.45
Mother's ART facility				
Outside delivery facility	Ref			
At delivery facility	0.93	0.31 – 2.76	0.93	0.31 – 2.70

Table 2: Comparison of complete case analysis and multiple imputation model estimates for the HIV testing model

Term	MICE estimate	CI	GLM estimate	CI
Intervention phase				
Pre-intervention	Ref			
Post-intervention	4.36	1.07 - 17.86	4.36	1.21 – 21.2
Maternal age	1.06	0.95 – 1.17	1.06	0.96 – 1.18
Mother's ART facility				
Outside delivery facility	Ref			
At delivery facility	4.63	1.38 – 15.49	4.63	1.44 – 16.2

In general, the estimates from the two approaches were largely consistent, suggesting that the conclusions were not majorly affected by the small departures from the MCAR assumption.

Model assessment

Key model assessment statistics indicated good fit. We did not find evidence of collinearity in the fitted models, with values clustered around 1. Hosmer-Lemeshow tests of goodness of fit also indicated that the models fitted well to the data ($p = 0.12$ for the enrolment model and $p = 0.42$ for the HIV model)
